# Beyond symptomatic diagnosis of mild traumatic brain injury

**DOI:** 10.2217/cnc-2023-0005

**Published:** 2023-05-22

**Authors:** Alice Lux Fawzi, Christian Franck

**Affiliations:** 1 Department of Mechanical Engineering, University of Wisconsin-Madison, Madison, WI 53706, USA

**Keywords:** asymptomatic, concussion, diagnosis, mild TBI, sub-concussive, symptoms, treatment

## Abstract

It is commonly assumed that there is no brain injury if there are no noticeable symptoms following a head impact. There is growing evidence that traumatic brain injuries can occur with no outward symptoms and that the damage from these injuries can accumulate over time resulting in disease and impairment later in life. It is time to rethink the role that symptoms play in traumatic brain injury and adopt a quantitative understanding of brain health at the cellular level to improve the way we diagnose, prevent, and ultimately heal brain injury.

## Tweetable abstract

TBI diagnosis and treatment based on symptoms are misguiding efforts to identify, prevent and ultimately heal brain injury. It is time to adopt a quantitative cellular understanding of TBI to detect all brain injuries irrespective of symptoms.

Traumatic brain injuries (TBIs) are a major global health challenge with a substantial societal burden. There is no cure, and many fundamental questions remain unanswered. An incomplete understanding of the molecular mechanisms at the root of injury serves as a poor foundation for clinical practice, leading to improper diagnoses and treatments that inadequately aim to manage symptoms, without curing the root cause of the disease.

Mild traumatic brain injury (mTBI), which umbrellas the symptoms that define concussion, describes the disrupted brain function following a head impact, violent motion, or blast exposure. The designation of *mild* distinguishes from moderate and severe TBI which frequently include focal injury and always result in loss of consciousness. In contrast, the damage associated with mTBI is diffuse and can disrupt normal brain function without any discernable outward signs of physical injury [1]. More than 90% of TBIs diagnosed in hospital are mild yet for half of these, normal function is not regained 6-months after injury [2]. Within mTBI, the lowest severity impacts are categorized as “sub-concussive” and most people never seek treatment despite findings that these impacts impair neurocognitive and neurophysiological function in the short term [1,3] and may precipitate neurodegenerative disease and neurocognitive disorders in the long term [4–7].

There is an excellent understanding of how much load a bridge or an airplane wing can bear without being damaged because the failure modes of steel, aluminum and concrete are well understood. This material failure model has been effectively applied to the body to predict how much force bones, like the skull, can withstand before fracture. In mTBI, the soft tissue within the skull deforms, disrupting brain function via damage at several scales – neural cells, cellular networks, and tissues. At present, there are two principal modes of brain injury. The first, *primary injury*, occurs when the magnitude of deformation of the brain tissue exceeds the threshold of what the cells or their networks can physically withstand, and cells or their networks are physically damaged [8,9]. For the second, a combination of deformation rate and magnitude initiate *secondary injury*, a term describing a complex series of biochemical reactions inside the cell that occur in the hours or days after impact, often leading to permanent impairment or cell death [8,10]. Part of this impairment features structural and molecular changes that can have debilitating effects later in life. One such example is the hyper-phosphorylation of the microtubule-associated tau protein, a key structural protein found in the axons of neurons associated with several neurodegenerative diseases such as chronic traumatic encephalopathy (CTE), dementia and Alzheimer's disease, poised to appear years after the brain is injured [4–7].

Following a head impact, there is currently no way to directly evaluate the health of neurons in a living person. Standard MRI or CT can only identify large scale structural abnormalities such as focal brain injuries, not the health of individual cells or networks. Without clinical methods sensitive to brain health directly at the scale of injury, symptoms, the downstream effects of mTBI, have been the only means available to identify injury in a living human. Consequently, symptoms have, by default, become accepted as the *definition* of injury instead of the *result* of injury, leading to the incorrect conclusion that a person without acute symptoms following a head impact is not injured. This definition is problematic considering that accumulation of minor impacts, each with no apparent symptoms, can lead to severe long-term consequences. For example, a 2014 study used fMRI to look at the brains of high school football players with no clinically observable signs of concussion. These players showed brain function impairments comparable to or exceeding those of teammates diagnosed with concussions [1]. Other studies on athletes playing soccer, rugby and hockey have yielded similar results [11,12]. The mTBI evaluations administered at the sideline in sport or in a standard doctor's visit are not sensitive enough to detect low severity injuries. Refined thinking about the role of symptoms reveals and motivates the need for better diagnosis and supports the conclusion that *concussion* is not a meaningful measure of brain injury.

Decoupling symptoms from the definition of injury and using deterministic measures of brain health will establish mTBI as a description of the whole spectrum of diffuse brain injury severity from asymptomatic up to moderate TBI. There have been numerous efforts to define the relationship between head motion and injury with the goal of establishing a more quantitative, deterministic link to injury and to identify injury thresholds to set safe levels of exposure for injury prevention. These efforts typically instrument athletes' heads with motion sensors, then measure the head motion from impacts through the normal course of sport. When a head impact occurs, the sensors measure the kinematics of the head, and the impact is categorized as “injurious” or “non-injurious” based on the symptoms of the impacted individual evaluated at the sidelines. For the impacts that cause obvious symptoms, this method is inadequate because there is variation in presentation of symptoms between individuals based on risk factors including history of head impacts. Conversely, the head impacts without obvious symptoms are interpreted as safe when there may be asymptomatic or sub-symptomatic damage that is not detected. When the outcomes from these studies are used as the basis for safe exposure recommendations or standards for protective equipment such as helmets, the research community gives the public a false promise of safety.

New methods *can* effectively detect subtle symptoms, biomarkers or brain changes following an injurious exposure. Eye-movement testing is emerging as a way to detect changes in attention and working memory resulting from low severity exposures [11]. A product using fluid biomarkers in samples of blood serum to check for brain injury has already been approved. Advanced brain imaging techniques can detect some degree of change in the structure or function of injured brains in a research setting [3,13]. Methods to assess metabolic irregularities [14] or changes in blood flow after injury [15] and even directly assess the function of neurons in living brains through the scalp and skull are in development. This exciting work not only gives hope for breakthrough in detecting mTBI but also in understanding the complicated chemo-mechanical-electrical complexity at the root of diffuse brain injury.

In the laboratory, new efforts merging science, engineering, and medicine are probing the direct causes of injury using high fidelity *in vitro* models that retain the essential complexity of the brain but allow more detailed characterization of injury than is possible in humans. Electrically active neural cells can be grown in the lab and deformed at controlled rates to simulate brain tissue strain during a head impact. The response of the cells over time reveals which conditions lead to cell death via primary or secondary injury, ultimately defining an injury threshold [9,10]. These experiments inform computer models at multiple scales. Sub-cellular models link deformation with disruptions in the cell's structure and function, showing how secondary injury is initiated or which structures fail first during primary injury [16]. This insight is applied to developing secondary injury interventions aimed at mitigating and preventing cell death [17]. At a much larger scale, whole brain models predict how brain tissue deforms in a simulated impact, applying the experimental cellular injury threshold to discern where and to what extent deformation is beyond what the cells and networks in the brain can withstand [18]. These whole brain models are emerging as one of the best tools available for understanding how head motion causes injury.

Symptom-based definitions of injury have been used to set safety guidelines and equipment specifications for sport, transportation, and military operations. The thresholds girding these measures are undoubtedly set too high to prevent mTBI. As evidence, contact and collision-sport athletes without persistent concussion symptoms develop neurodegenerative disease at high rates. It is urgent to apply the latest insights to new guidelines and equipment to protect athletes, soldiers, and the public at large. Quantitative and deterministic brain models can be applied now to direct the design and testing of protective headgear to better prevent or reduce injury [19].

A new, quantitative understanding of the root cause of mTBI, based on the biophysics of cellular injury is the deterministic foundation needed to predict, prevent, and ultimately heal brain injury. Thanks to the exciting convergence of new breakthrough technologies including advanced microscopy and artificial intelligence with interdisciplinary research teams comprised of experts from the life, physical and engineering sciences, a new era in TBI research is arriving. We need to be ready to leave behind a symptom-only view of mTBI diagnosis and finally resolve crucial questions that have been stymied in debate for decades.
